# The Diagnosis, Pathology, and Treatment of the Diseases of the Chest

**Published:** 1846-06

**Authors:** 


ART. VI.—*The Diagnosis, Pathology, and Treatment of the Diseases of the Chest.* By W. W. GERHARD, M.D., Lecturer on Clinical Medicine to the University of Pennsylvania; one of the Physicians to the Pennsylvania Hospital, &c. Second edition, revised and enlarged. Philadelphia: Ed. Barrington & Geo. D. Haswell. 1846. 8vo. pp. 288.

The author of this volume has attained among the medical writers and practitioners of this country, an enviable distinction, and a work from his pen on the *Diseases of the Chest* will be accredited as the highest authority on that subject. Dr. Gerhard has had the most ample opportunities of studying disease in a large hospital, and for many years he has devoted special attention to thoracic complaints. As indicated by the title-page, this is the second edition of his work, important additions having been made to it, the results of his unwearied devotion to hospital and private practice. It forms, now, a very complete monograph on pectoral diseases, embracing not only the physical and general signs, but the treatment also of such affections. The work stands in no need of any commendation from us, having already by public opinion been assigned a place among our best indigenous medical productions.

